# Real-Time Detection of Hemothorax and Monitoring its Progression in a Piglet Model by Electrical Impedance Tomography: A Feasibility Study

**DOI:** 10.1155/2020/1357160

**Published:** 2020-02-27

**Authors:** Lin Yang, Chao Zhang, Wenbo Liu, Hang Wang, Junying Xia, Benyuan Liu, Xuetao Shi, Xiuzhen Dong, Feng Fu, Meng Dai

**Affiliations:** ^1^Department of Aerospace Medicine, Fourth Military Medical University, Xi'an, China; ^2^Medical Engineering Section, General Hospital of Shenyang Military Region, Shenyang, China; ^3^Xijing hospital, Fourth Military Medical University, Xi'an, China; ^4^Department of Biomedical Engineering, Fourth Military Medical University, Xi'an, China

## Abstract

Hemothorax is a serious medical condition that can be life-threatening if left untreated. Early diagnosis and timely treatment are of great importance to produce favorable outcome. Although currently available diagnostic techniques, e.g., chest radiography, ultrasonography, and CT, can accurately detect hemothorax, delayed hemothorax cannot be identified early because these examinations are often performed on patients until noticeable symptoms manifest. Therefore, for early detection of delayed hemothorax, real-time monitoring by means of a portable and noninvasive imaging technique is needed. In this study, we employed electrical impedance tomography (EIT) to detect the onset of hemothorax in real time on eight piglet hemothorax models. The models were established by injection of 60 ml fresh autologous blood into the pleural cavity, and the subsequent development of hemothorax was monitored continuously. The results showed that EIT was able to sensitively detect hemothorax as small as 10 ml in volume, as well as its location. Also, the development of hemothorax over a range of 10 ml up to 60 ml was well monitored in real time, with a favorable linear relationship between the impedance change in EIT images and the volume of blood injected. These findings demonstrated that EIT has a unique potential for early diagnosis and continuous monitoring of hemothorax in clinical practice, providing medical staff valuable information for prompt identification and treatment of delayed hemothorax.

## 1. Introduction

Hemothorax is a serious medical condition characterized by the accumulation of blood within the pleural cavity [1, 2]. It is a frequent consequence of open or closed chest injury that occurs in approximately 60% of all polytrauma cases and has been the second most common traumatic injury [3, 4]. Whilst it is reported that 25% of trauma death was due to chest trauma, hemothorax is potentially a fatal cause with high mortality [5–7]. Hemothorax can be dangerous if left untreated. Prolonged retention of blood within a pleural space may initially have a fibrotic reaction that prevents adequate lung expansion leading to atelectasis and ventilation/perfusion mismatch [8, 9]. Moreover, the retained blood may also be contaminated by bacteria and cause empyema (even bacteremia and septic shock) [10, 11]. However, clinical studies suggest that early identification and treatments of hemothorax, such as controlling the bleeding and evacuating the hematoma from the pleural space, can produce evidently favorable outcome [12–14]. Therefore, early detection and prompt treatment of hemothorax are of outmost importance to improve the prognosis of patients.

According to the time of occurrence, hemothorax can be classified into acute or delayed cases. In the acute case, hemothorax could be easily identified on an upright chest radiography, ultrasonography, or CT scan immediately after trauma or at the time of admission [14]. However, in the delayed case, especially for closed traumatic condition, the current imaging techniques cannot readily capture the initial bleeding within pleural cavity. It is because hemothorax may not occur until hours or even days after injury, and these examinations are often carried out till patients manifest noticeable symptoms [15, 16], including chest pain and difficulty in breathing [14, 17]. If treated too late, delayed massive hemothorax could be life-threatening [13, 18]. Hence, for those patients with high-risk factors of delayed hemothorax, such as multiple or displaced rib fractures [19, 20], a portable, noninvasive, and radiation-free medical device is highly required for long-term and real-time monitoring of hemothorax for early detection [21].

Electrical impedance tomography (EIT), a relatively new computed tomography technology, can produce cross-sectional images of electrical impedance distributions inside the human body. In EIT, safe electrical currents are injected into the human body to induce boundary voltages, which result from internal tissues of the body and are measured through an array of surface electrodes attached to a chosen imaging domain such as the chest. Then EIT images are reconstructed from the measured boundary voltages using a certain image reconstruction algorithm [22]. Compared with X-ray, ultrasonography, and CT, EIT has the advantages of small size, low cost, noninvasiveness, no radiation, and high temporal resolution, which make it a promising imaging technique in many biomedical applications, especially in real-time monitoring [23]. Up to the present, EIT has been widely studied on the application of monitoring lung ventilation and perfusion [24–27]. In addition, due to the significant difference in electrical impedance property between the blood and other human tissues [28, 29], EIT has a great potential in long-term and continuous detection of occurrence and progression of internal hemorrhage in human organs, including hemothorax.

To date, several studies have reported the feasibility evaluation of using EIT to monitor internal hemorrhage in real time. Xu et al., Manwaring et al., Dowrick et al., Sadleir et al., and Dai et al. established different animal models of intracranial hemorrhage (ICH), including intraparenchymal hemorrhage (IPH), subarachnoid hemorrhage (SAH) and intraventricular hemorrhage (IVH). They found that EIT could accurately reflect the real-time impedance change caused by ICH, even though the blood volume was much smaller than that of the brain, as in the case of the IPH pig model with 5 ml blood injection [30–34]. Moreover, Shuai et al. explored the ability of EIT to monitor intraperitoneal hemorrhage in a pig model and observed that the intraperitoneal blood volume changes could be continuously identified by the observation of EIT images throughout the bleeding progression [35]. In another study, You et al. successfully applied EIT to dynamically monitor retroperitoneal bleeding in a renal trauma patient over a period of 9 h [36]. In brief, previous studies have demonstrated that EIT has unique advantages in long-term and continuous monitoring of internal hemorrhage in different organs. To our knowledge, however, there is no study that evaluate the feasibility of continuous monitoring of hemothorax by EIT.

Accordingly, in this study, we first established hemothorax models by injecting autologous blood into the pleural cavity of pigs. Second, lung EIT data was continuously collected throughout the whole course of blood injection before being processed by a digital filtering method to obtain independent impedance change caused by hemothorax for EIT image reconstruction. Finally, we quantitatively analyzed the relationship between the impedance change reflected by EIT images and the volume of the blood injection.

## 2. Materials and Methods

### 2.1. Preparation of Animals

All experiments in this study were approved by the Institutional Animal Care and Use Committees of the Air Force Medical University, Xi'an, Shaanxi, China.

Eight piglets aged 2 months (weight, 13 ± 0.5 kg; four females and four males) were obtained from the experimental animal center and bred on site. In order to avoid the backflow of food in the stomach that would block the respiratory tract after anesthesia, the piglets were deprived of food and water for 12 h before the experimental procedures. All the experiments were performed under a relative stable ambient temperature (27 ± 1.3°C), and the core temperature of the animals was measured using a rectal thermistor probe. The subjects were initially anesthetized via intramuscular injection of atropine (0.05 mg/kg) to reduce respiratory secretions. After sedation, pentobarbital was administrated through intraperitoneal injection (30 mg/kg) to achieve a deep level of anesthesia for further surgical procedures. Pancuronium was used to maintain muscle paralysis (0.2 mg/kg, repetitive 0.1 mg/kg). Next, the thoracic skin within a range of 15 cm behind the piglets' forelimbs was shaved and cleansed to prepare EIT electrode attachment.

To ensure that the piglets maintain normal breathing throughout the experiment, an animal ventilator was used. The procedures of tracheal intubation are as follows: First, the piglets were fixed in the supine position so that the head and neck were lower than the body; then a laryngoscope was used to pick up the piglets' tongue and the epiglottis to find the tracheal opening. Second, a tracheal cannula was inserted into the trachea with an insertion depth of approximately 20-25 cm during inhalation, and the tracheal intubation was considered to be successful if a fog appeared. Afterwards, the piglets were fixed in the prone position. The parameters of the ventilator were set to a respiratory rate of 25 beats/min and a tidal volume of 10 ml/kg.

### 2.2. Establishment of the Hemothorax Model

**Figure 1:**
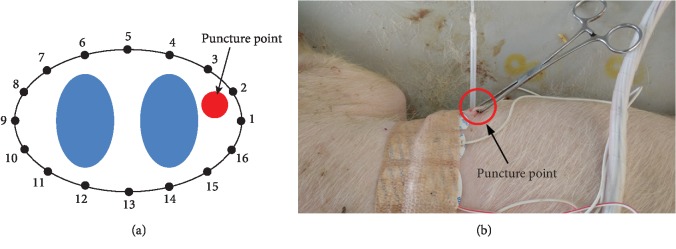
Puncture point in the establishment of the hemothorax model. (a) Schematic diagram (indices 1-16 represent EIT electrodes 1-16). (b) Experimental photo.

Autologous blood injection was employed to establish the hemothorax model. To prepare the blood injection, 80 ml of blood was collected from the femoral artery using a 100 ml syringe, in which heparin was aspirated in advance to prevent blood coagulation. Then 60 ml of the heparinized blood was aspirated into another 100 ml syringe connected to a puncture needle through a drainage tube, which was fixed to a microinjection pump (AT-5803) operated at the rate of 600 ml/h. Using a scalpel, a small incision (about 0.5 cm in length) was carefully made between the fifth and sixth ribs without injuring the parietal pleura (Figure 1). With reference to thoracic closed drainage, the puncture needle was slowly inserted through the incision to the extent when there was a clear perception of breakthrough, indicating the entering of the puncture needle into the pleural cavity. A pair of hemostatic forceps was applied to stop the bleeding rapidly, and a Vaseline seal was used to prevent air leakage. Finally, the microinjection pump was activated to provide continuous blood injection.

### 2.3. EIT System Protocol

#### 2.3.1. EIT Electrode Application

**Figure 2:**
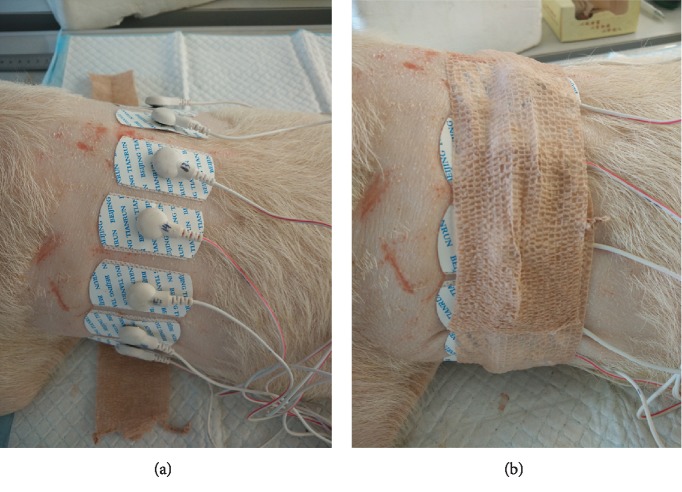
EIT electrode application. (a) Electrode placement. (b) Electrode consolidation using a self-adhesive bandage.

The EIT electrode system consisted of 16 disposable Ag+/Ag+Cl− electrodes (Shanghai Shenfeng Medical & Health Articles Co., Ltd., Shanghai, People's Republic of China) with 10 mm diameter. After the shaved skin of all piglets was cleansed with alcohol, the electrodes were attached to the skin 5 cm above the xiphisternum, equally spaced around the thorax. In order to enhance the stability of the electrodes, a self-adhesive bandage (McDavid-4575, Bellwood, USA) with a length of 1.5 times the thorax circumference was employed to wrap around the thorax twice (Figure 2).

#### 2.3.2. EIT Data Acquisition

EIT data acquisition was performed using a portable EIT system (PEIT4) with high precision, as well as high acquisition speed. The system is specifically designed for lung imaging by our group, which can operate at 100 kHz and output current of 1 mA constantly. The measurement accuracy is better than 1‰, and the acquisition rate can be up to 12 frames/s [37]. Despite the small volume (10∗5∗2 cm^3^), this system can work continuously for more than 10 h using a lithium battery. To increase the sensitivity to changes in impedance caused by central lung regions and maintain a good signal-to-noise ratio, the protocol of opposite excitation-adjacent measurement protocol was adopted in EIT data acquisition [38].

**Figure 3:**
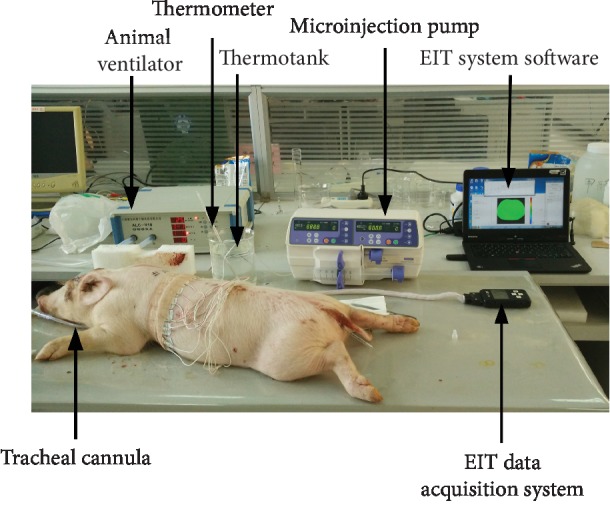
View of EIT monitoring of simulated pulmonary hemothorax on a piglet.

For data comparison, a control group consisting of EIT data acquired 5 min before blood injection and the corresponding reconstructed images were obtained. After the commencement of blood injection, EIT data was continuously collected for more than 6 min (Figure 3).

#### 2.3.3. EIT Data Processing

The EIT data was continuously acquired during the subjects' normal respiration throughout the entire process of blood injection. Therefore, the EIT data reflected the impedance change caused by both blood injection and normal breathing. To analyze the relationship between the EIT data and blood injection, we used a digital filtering technology to obtain independent impedance change induced by blood injection based on the fact that breathing had obvious periodicity whereas blood injection did not. The steps of EIT data processing were as follows: First, the power spectra of boundary voltages of all channels (these boundary voltages composed the EIT data) were calculated, respectively. Second, a 4-order low-pass Butterworth filter with a stop band boundary frequency of 0.15 Hz was designed and used to process the EIT data to obtain the independent signal of blood injection because the frequency corresponding to breathing was approximately 0.4 Hz (the frequency of the ventilator was set to 25 beats per minute). Third, the independent signal corresponding to breathing can be gained by subtracting the independent signal of blood injection from the raw EIT data.

To exhibit the changes of the EIT data, the total boundary voltage variation (TBVV) was used, which was calculated using the following formula:

(1) $$\begin{array}{c} {\text{TBVV}}_{t}=\frac{{\text{TBV}}_{t}-{\text{TBV}}_{\text{ref}}}{{\text{TBV}}_{\text{ref}}}\cdot 100\%, \end{array}$$

(2) $$\begin{array}{c} \text{TBV}=\overset{16}{\underset{i=1}{\sum }}\overset{16}{\underset{j=1}{\sum }}v_{i,j}\;\left(j\neq 1,8,9,16\right), \end{array}$$

where TBV_*t*_ and TBV_ref_ denote the total boundary voltage (TBV) at time point *t* and the reference time point. *v*_*i*,*j*_ is the boundary voltage of the measurement electrode pair *j* when the current is excited through the electrode pair *i*. In practice, the voltage difference caused by electrode-skin contact impedance is much larger than that resulted from the impedance of internal body tissue [39]. Moreover, the contact impedance often changes with time due to various inevitable factors such as perspiration, temperature change, patient movement, and manipulations by clinical staff [40]. Additionally, it is difficult to precisely measure the electrode-skin contact impedance of exciting electrodes [41]. Thus, the measured voltages are deducted when they are measured on electrode pairs that comprise one of the exciting electrodes, i.e., *j* ≠ 1, 8, 9, 16.

#### 2.3.4. EIT Image Reconstruction

EIT difference images were reconstructed with the damped least-squares (DLS) algorithm, whose effectiveness for monitoring internal bleeding has been validated by animal and human experiments [42]. The reconstruction matrix was computed from a 2D circular model with uniform electrical impedance distribution and stored in advance. The image reconstruction process was as follows:

(3) $$\begin{array}{c} \Delta \rho ={\left[S^{\text{T}}S+\lambda L\right]}^{-1}S^{\text{T}}\cdot \Delta v=B\cdot \Delta v, \end{array}$$

where Δ**ρ** represents the impedance change vector (image) between two time points, **S** is the linearized sensitivity matrix, **L** denotes the regularization matrix which is calculated based on a smoothness assumption of **L** = diag(**S**^T^**S**) [43], *λ* is the regularization parameter which is usually computed using the L-curve method (here, *λ* = 0.1), **B** is the reconstruction matrix **B** = [**S**^T^**S** + *λ ***L**]^−1^**S**^T^, and Δ**v** is the boundary voltage variation vector between two time points.

(4) $$\begin{array}{c} \Delta v=\frac{v_{t_{x}}-v_{t_{\text{ref}}}}{v_{t_{\text{ref}}}}, \end{array}$$

where **v**_*t*_ref__ is the boundary voltage at the reference time point and **v**_*t*_*x*__ represents the boundary voltage acquired at another time point.

### 2.4. EIT Data Analysis

In order to quantitatively analyze the impedance change in EIT images resulting from pH, we used regional impedance variation (RIV) which measured the degree of impedance change of the region where blood was injected. RIV is calculated as follows:

(5) $$\begin{array}{c} \text{RIV}=\frac{\sum_{i=1}^{N}\Delta \rho_{\Omega_{i}}\cdot S_{\Omega_{i}}}{\sum_{j=1}^{M}S_{\Gamma_{j}}}, \end{array}$$

where *Ω* represents the region of blood injection; Δ*ρ*_*Ω*_*i*__ and*S*_*Ω*_*i*__ are the reconstructed impedance and the area of the *i*th element within the region of blood injection, respectively, representing the area of the *j*th element within the whole imaging region; and *N* and *M* denote the number of finite elements in the region of blood injection and the total number of finite elements in the whole imaging region.

To determine the region of blood injection in the reconstructed image, the threshold value strategy was employed. First of all, the maximum reconstructed value *ρ*_max_ was found by comparing the reconstructed values of all finite elements in the EIT images. Subsequently, the area consisting of all finite elements with a reconstructed value larger than 10% of *ρ*_max_, was considered the region where blood was injected.

In this study, the IBM SPSS Statistics for Windows (version 22; IBM Corporation, Armonk, NY, USA) was used to perform statistical analysis. The comparisons of the difference in RIV 5 min before blood injection and after 10 ml, 20 ml, 30 ml, 40 ml, 50 ml, and 60 ml of blood was injected was carried out with one-way analysis of variance (ANOVA). The post hoc test was used, and *p* < 0.05 was deemed statistically significant.

Furthermore, to study the relationship between impedance change and blood injection, linear regression analysis between RIV and time (namely, the amount of the injected blood) was performed for both individual subjects and the pooled data.

### 2.5. Validation of Hemothorax Model

After the experiment was completed, the piglet was sacrificed by administering a pentobarbital overdose. According to the position of the puncture needle, the small incision (0.5 cm in length) made to insert the puncture needle was further enlarged to expose the pleural cavity at the site of the blood injection. This allows us to directly observe the distribution of the injected blood.

## 3. Results

During all the experiments, the subjects maintained stable respiration and body temperature. During the validation of the hemothorax model in one animal, we found that blood was injected into the lung tissue; therefore, one more animal was added. Eight sets of EIT data were successfully collected.

### 3.1. The Hemothorax Model

**Figure 4:**
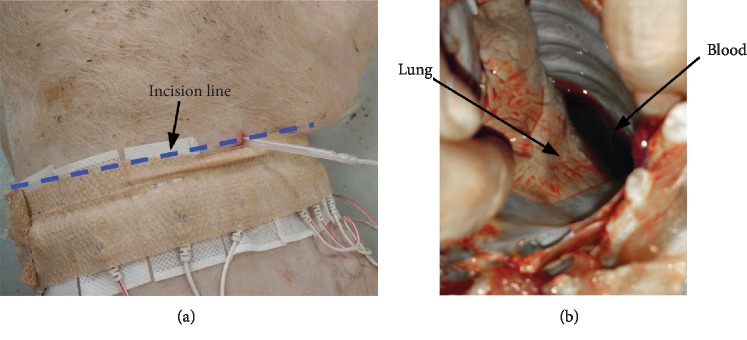
The established hemothorax model. (a) Incision line to expose the pleural cavity. (b) Anatomical structure of the hemothorax model.

Figure 4(a) shows the incision line, along which the pleural cavity was exposed.  Figure 4(b) shows that the blood was found in the pleural cavity, indicating that the hemothorax model was successfully established.

### 3.2. EIT Imaging in a Normal Respiratory Circle

**Figure 5:**
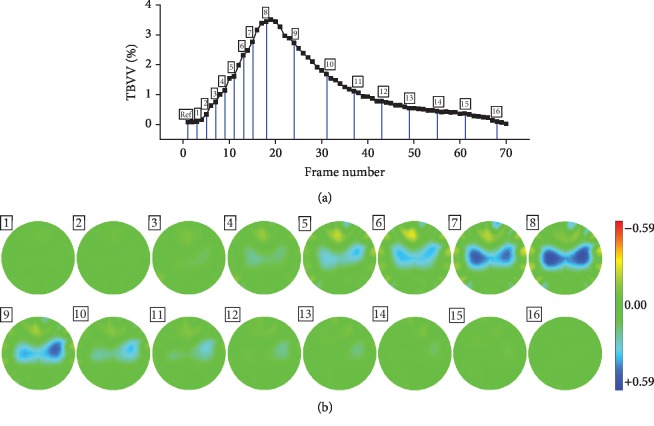
EIT imaging in a normal respiratory circle. (a) Total boundary voltage variation (TBVV). (b) EIT images. In the EIT images in this study, the red and blue areas represent a regional impedance reduction and increase, respectively.

Figure 5 shows the boundary voltage and the EIT images in a normal respiratory circle. As shown in Figure 5(a), TBVV gradually increased during inhalation and decreased during exhalation. Similarly, the impedance within the lung area gradually increased during inhalation and decreased during exhalation, which is reflected in the EIT images shown in Figure 5(b). These results validated the reliability and accuracy of the EIT system used in this study.

### 3.3. Separation of Respiratory Impedance and Blood Injection Impedance by Filtering

**Figure 6:**
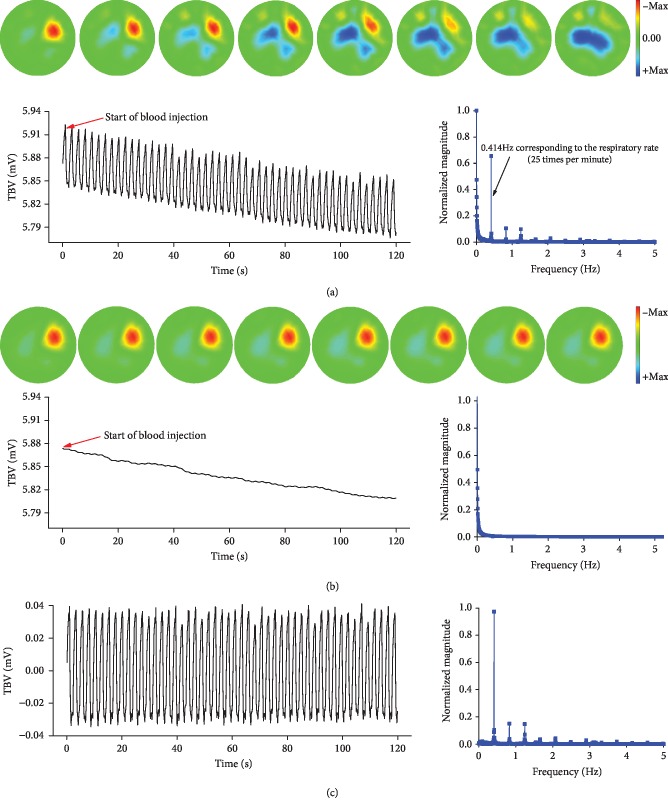
Separation of respiration and blood injection by filtering. (a) Raw EIT images during inhalation when using the data at the end of exhalation as the reference frame, raw total boundary voltage (TBV) during blood injection, and raw power spectra. (b) EIT images, TBV, and power spectra corresponding to blood injection obtained through a 4th-order low-pass Butterworth filter with a cutoff frequency of 0.15 Hz. (c) TBV and power spectra corresponding to respiration obtained by subtracting TBV caused by blood injection from the raw TBV.

Figure 6 shows the procedure of separating the impedance caused by blood injection from that resulting from respiration. As shown in Figure 6(a), when using the EIT data at the end of exhalation as the reference frame, the impedance change caused by blood injection was gradually covered by that resulting from inhalation in the EIT images. Additionally, TBV not only changed with respiration but also decreased with the volume increase of the injected blood. From the power spectra of TBV, it is seen that TBV contained two major factors, one of which is the signal at 0.4 Hz corresponding to the respiratory rate (25 beats per minute) and the other is the signal below 0.1 Hz corresponding to the blood injection rate (if blood injection was considered a signal that had been changing very slowly).

Figure 6(b) shows that after filtering through a 4th-order low-pass Butterworth filter with a cutoff frequency of 0.15 Hz, only the impedance change caused by blood injection was displayed in the EIT images. Moreover, the curve representing TBV also reveals a direct and exclusive correlation with blood injection over time, which is, again, demonstrated by the power spectra.

Figure 6(c) shows the independent TBV and power spectra corresponding to respiration, which was obtained by subtracting TBV caused by blood injection from the raw TBV.

### 3.4. EIT Imaging of the Blood Injection Procedure

**Figure 7:**
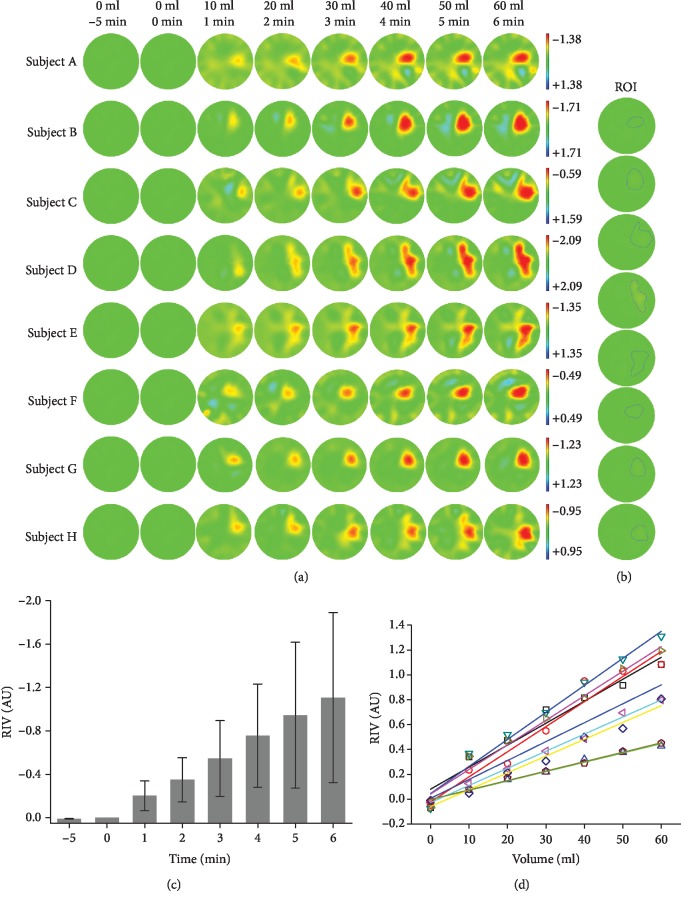
EIT imaging of the hemothorax procedure in the piglets. (a) A series of reconstructed EIT images of the eight subjects before and during the entire course of blood injection. (b) The individualized region of interest (ROI) in each of the subjects where blood was injected. (c) The statistical comparison of regional impedance variation (RIV) for all the subjects before and after blood injection. (d) The linear regression analysis between RIV in EIT images and the volume of blood injection for all eight piglets as well as for the pooled data. Each line represents a single piglet marked with a unique color and a symbol. The bold blue line denotes the mean relationship between RIV and blood volume for the eight piglets.

Figure 7(a) shows the variation in the EIT images for 5 min before blood injection as well as during the entire course of blood injection for all eight piglets. Overall, the trend of impedance changes for all the piglets is similar. Before injecting blood, no significant impedance change was observed. After 10 ml of blood was injected, the impedance of the region of injection decreased. As the amount of blood increased, the impedance variation gradually became obvious, exhibiting a growing magnitude of the impedance change and an enlarging area. After the blood injection was completed (60 ml of blood), the impedance change reached its maximum with the greatest amplitude and the largest area. However, the degrees of impedance change of all the subjects were different, showing a variety of amplitudes (indicated by color bars) and various areas.

Figure 7(b) defines the ROIs where blood was injected for all the subjects, through which the regional impedance variations (RIV) caused by blood injection were calculated.

In Figure 7(c), a significant difference in RIV can be observed after only 10 ml of blood was injected by comparing the RIV for 5 min before blood injection (0.041 ± 0.027 (mean ± SD)) with that for 1 min after blood injection (-0.202 ± 0.136, *p* = 0.0378). Besides, with more blood being injected (20 ml, 30 ml, 40 ml, 50 ml, and 60 ml), the RIVs also showed a significant difference from the RIV before blood injection (*p* < 0.00276).

As given in Figure 7(d) and Table 1, the linear regression indicates that RIV favorably predicted the volume of blood injection for each piglet with the determination coefficient *R*^2^ range of 0.947-0.995 (*p* < 0.001). This was similar for pooled data from all eight piglets (*R*^2^ = 0.669; *p* < 0.001).

## 4. Discussion

### 4.1. Summary and Explanations of Results

In clinical practice, it is highly essential to develop new methods for early detection of delayed hemothorax in order to produce favorable outcomes, because contemporary medical imaging modalities, such as chest X-ray, CT, and ultrasonography, cannot perform long-term and real-time monitoring of chest trauma patients at high risk of delayed hemothorax. In this study, we explored the feasibility of using EIT to detect hemothorax in an animal model. The results suggested that EIT was able to sensitively identify hemothorax with volume as small as 10 ml and its location. In addition, EIT was able to carry out real-time monitoring of the hemothorax development (from 10 ml up to 60 ml) based on a desirable linear relationship between the impedance change in EIT images and the volume of the injected blood.

In all the experiments, we continuously collected EIT data over a period of 11 min, including 5 min before blood injection and 6 min of blood injection. During the first 5 min, there was no significant impedance change in EIT images for all the subjects (Figure 7(a)), and RIV indicated that the impedance in the area of blood injection showed no dramatic changes compared with that after blood injection (Figure 7(c)). These results demonstrate the reliability of the EIT system used in this study. Over the subsequent 6 min, the impedance in the blood injection area gradually decreased as more blood was injected, due to the fact that the electrical resistivity of blood (1.43 Ω·m) is much lower than that of lung tissues, e.g., a deflated lung (3.82 Ω·m) and an inflated lung (9.74 Ω·m) [44]. These results are largely in agreement with the reports from Hahn et al., in which EIT was employed to record the impedance change in a pig model of hemothorax established by injecting Ringer solution into the pleural space of piglets [45]. Additionally, the average TBV resulted from blood injection increased by 0.86-1.52% during the process, further demonstrating the feasibility and the high sensitivity of EIT to detect hemothorax.

In the analysis of the EIT images, we adopted an index, referred as RIV, to evaluate the relationship between impedance change and blood injection. For each subject, RIV decreased almost linearly with continuous blood injection (*R*^2^ > 0.947) and was significantly correlated with the blood volume (*p* < 0.001). Moreover, for all the subjects, *R*^2^ can be up to 0.669, which further exhibits the favorable linear correction between impedance change and blood volume. Thus, RIV may be regarded as a sensitive and practical metric to reflect hemothorax development. A future work is expected to find more metrics for EIT images apart from RIV to form a reasonable index system to comprehensively quantify blood injection.

### 4.2. Technical Considerations and Limitations

In this study, we collected the EIT data from each subject during two time periods, which were 6 min of blood injection and 5 min prior to that course. Throughout the entire data collection process, the animal kept breathing normally. Therefore, the acquired EIT data contained impedance information on both respiration and blood injection. In order to quantitatively analyze the impedance change solely caused by blood injection, we designed a 4-order low-pass digital filter to separate the two kinds of impedance change, considering the significant periodicity of breathing other than blood injection. Our results suggest that the digital filter we designed is valid. Similarly, in the field of lung EIT research, many efforts had already been made to separate different kinds of signals, such as cardiac and respiratory signals. For instance, Deibele et al. proposed a principle component analysis- (PCA-) based method to separate the respiratory and the perfusion-related signals, in which the template functions for pulmonary and cardiac component generated by PCA and frequency domain filtering were fitted into the input signals [46]. Rahman et al. used independent component analysis (ICA) to extract cardiac-related as well as respiratory-related impedance change in spontaneous breathing subjects [47]. In the future, we will compare these previously proposed methods with the digital filter method in the extraction of breath-related and hemothorax- (through blood injection) related impedance information.

In this study, the reconstruction model we used is not perfect. Firstly, we used a circular model with homogeneous impedance distribution to simplify the piglets' chest because the CT or MRI images of the chest of all the animals were not collected. This inaccuracy of the reconstruction model was supposed to induce some errors in the reconstructed images including errors of position and the anomaly (hemorrhage) shape, although our results suggest that the EIT images are able to sensitively reflect the progression of blood injection [48]. Secondly, as currents flow in a 3D spatial distribution, the 2D reconstruction model we used may also give rise to location and shape errors even though 2D EIT images can reflect the impedance information near the electrode plane. These imaging errors may have a certain degree of impact on the quantitative analysis of the EIT images, which is adverse to future clinical applications [49]. Consequently, future works, particularly human experiments, are needed to establish an individualized 3D chest reconstruction model to reduce the imaging error and improve the quality of EIT images in patients, which can be achieved by incorporating accurate geometry and realistic impedance distribution of the chest into the imaging reconstruction algorithm based on patients' CT or MRI images.

In order to establish a reproducible animal model of hemothorax, the autologous blood was treated with heparin to prevent coagulation before being injected into the pleural space. Thus, there exists a difference between the established hemothorax model and actual hemothorax. However, our results showed that EIT was capable of monitoring the change of blood within the pleural space, which demonstrated the feasibility of using EIT for real-time detection of hemothorax. In the future, we will improve the animal model by injecting nonanticoagulated autologous blood into the pleural space and further validate the ability of EIT to continuously monitor hemothorax.

### 4.3. Perspectives for Future Clinical Application

A hemothorax animal model was established in the present study by injecting blood into the pleural cavity of the piglets' lungs over a relatively short period of time (6 min), and the experimental conditions were strictly controlled in all cases. Hence, considerable factors affecting the quality of the collected EIT data were minimized. On the other hand, the EIT data mainly reflect the impedance changes caused by animal respiration and blood injection. However, in practical clinical cases, it is often required that real-time monitoring be implemented over an extended period of time in order to detect delayed hemothorax in time. This will exert many complicated influences on EIT data, such as perspiration or temperature change leading to the variation of electrode-skin contact impedance [40], as well as patient movement or manipulations by clinical staff resulting in corrupted data measurement [50]. These actual factors will severely reduce the quality of EIT data. Accordingly, it is imperative to study appropriate methods to improve the data quality before proceeding to clinical applications for hemothorax detection.

During long-term monitoring, perspiration, changes in temperature and humidity of the electrode-conductive gel-skin interface, electronics drift, and electrode contact change may often occur and inevitably cause a variation in the electrode-skin contact impedance [51]. Unfortunately, even a small change in electrode-skin contact impedance can obviously affect the accuracy of the measured EIT data, causing significant artifacts in EIT images. This is because both current excitation and boundary voltage measurement are carried out through the electrode-skin interface [52]. So far, electrode-skin contact impedance has been a main factor responsible for the influence on the quality of EIT data and image in long-time monitoring using EIT. In order to alleviate the effects of contact impedance, some novel reconstruction algorithms have been proposed with the complete electrode model (CEM), in which the contact impedance is introduced into the imaging reconstruction in an attempt to simultaneously recover both the contact impedance and the internal impedance of the human body. Boverman et al. used linear-algebraic methods in the inverse problem solving to reconstruct time-varying contact impedance and the interior impedance [53]. Demidenko tried to separately estimate the contact impedance by relying on the analytical solution with a Neumann-to-Dirichlet matrix [54]. Tarvainen et al. formulated EIT image reconstruction as one of the Bayesian estimations to compute the contact impedance [55]. The experiments corresponding to the above algorithms exhibited the possibility of significantly mitigating the effects of contact impedance. Additionally, Yang et al. compared the time property of contact impedance of 16 combinations (comprising 4 kinds of clinical electrode and five types of commonly used conductive gel) on ten volunteers in order to find the optimal combination for long-term monitoring. Their results indicated that the combination of Ag+/Ag+Cl− powder electrode and low viscosity conductive gel might be the best choice [56]. Therefore, future clinical studies on hemothorax detection should apply multiple methods to minimize the impacts of contact impedance on EIT images, including the use of specified image reconstruction algorithms and the selection of particular electrode and conductive gel.

Body movement interferences from patient movement or manipulations by clinical staff are common in prolonged EIT monitoring in clinical settings. It may cause electrode movement and complete or partial disconnection of the electrodes, leading to variation in measurement data and even remarkable data loss. To reduce body movement interferences, special imaging reconstruction algorithms and data preprocessing methods were designed. For electrode movement, Soleimani et al. developed a new approach in which both electrode movements and internal impedance change were both reconstructs, and the results showed a dramatic reduction in image artifacts caused by electrode movement as well as good reconstruction of the actual electrode movement [57]. Furthermore, for the case of complete electrode disconnection, Zhang et al. recently conceived a weighted correlation coefficient method to test multiple problematic electrodes and employed data from grey model predictions for compensatory processing [58]. Subsequently, for partial electrode disconnection, Zhang et al. proposed an online strategy based on wavelet decomposition to manage the EIT data from partially connected electrodes [59]. Consequently, in the future, approaches including special image algorithm and data preprocessing method to reduce the influence of body movement interferences are suggested to be used in clinical EIT application for delayed hemothorax detection.

### 4.4. Implications for Clinical Applications

In clinical settings, pathological fluid accumulation in the pleural cavity is a common symptom and it will severely affect pulmonary ventilation. Thus, evaluation of its impact by EIT is of great importance and several current studies have investigated this issue. Campbell et al. observed an increase in electrical resistivity on the affected chest side in patients with one-side pleural effusion from lung cancer during stepwise aspiration of pleural effusion [60]. Arad et al. obtained similar results when examining patients with pleural fluid before and after the removal of pleural effusion [61]. In addition, Alves et al. used EIT to evaluate lung reaeration, reventilation, and ventilator synchrony before and over one hour after a pleural aspiration [62]. Recently, Becher et al. concluded that EIT could be used to detect pleural effusion by analyzing the out-of-phase impedance between patients with and without a pleural fluid, as well as by comparing the impedance before and after drainage of an effusion [63]. The previous studies have fully demonstrated the reliability of using EIT to monitor the drainage of pleural effusion, namely, the decrease of fluid in the pleural cavity. Conversely, in this study, we explored the feasibility of using EIT to monitor the development of hemothorax, which is essentially the increase of fluid in the pleural cavity. In brief, this study and the previous studies have demonstrated the ability of EIT to detect the variation (decrease and increase) of pleural fluids, confirming the great potential of the EIT technique to assess pathological status and diseases related to pleural fluids.

## 5. Conclusions

In this study, we explored the feasibility and sensitivity of the use of EIT technique to early detect and continuously monitor the development of hemothorax on piglet hemothorax models established by injecting the blood into the pleural cavity. For clinical practice, more specific studies are needed that focus on the use of a realistic 3D chest reconstruction model, development of image reconstruction algorithms, and data preprocessing method. Despite that, our results in this study demonstrate that EIT has a unique potential for early diagnosis and continuous monitoring of hemothorax in patients with high-risk factors, providing medical staff valuable information for prompt identification and treatment of delayed hemothorax.

## Figures and Tables

**Table 1:** Linear regression analysis between regional impedance variation (RIV) in the EIT images and the volume of blood injected into the pleural cavity of each of eight piglets (P1-P8) to establish a hemothorax model. The *F* test was used to evaluate the established linear statistical model.

Subject	Determination coefficient (*R*^2^)	Regression coefficient	*p*
P1	0.947	-1.77 × 10^−2^	2.27 × 10^−4^
P2	0.955	-2.01 × 10^−2^	1.52 × 10^−4^
P3	0.993	-7.51 × 10^−3^	1.64 × 10^−6^
P4	0.978	-2.17 × 10^−2^	2.38 × 10^−5^
P5	0.995	-1.37 × 10^−2^	6.34 × 10^−7^
P6	0.976	-1.97 × 10^−2^	3.01 × 10^−5^
P7	0.980	-1.35 × 10^−2^	1.97 × 10^−5^
P8	0.994	-7.59 × 10^−3^	8.05 × 10^−7^
Pooled data	0.669	-1.52 × 10^−2^	1.41 × 10^−14^

## Data Availability

The data used to support the findings of this study are available from the corresponding author upon request.
